# circ-PTK2 (hsa_circ_0008305) regulates the pathogenic processes of ovarian cancer via miR-639 and FOXC1 regulatory cascade

**DOI:** 10.1186/s12935-021-01985-x

**Published:** 2021-05-25

**Authors:** San-Gang Wu, Ping Zhou, Jian-Xian Chen, Jian Lei, Li Hua, Yong Dong, Min Hu, Chen-Lu Lian, Li-Chao Yang, Juan Zhou

**Affiliations:** 1 Department of Radiation Oncology, The First Affiliated Hospital of Xiamen University, Xiamen, 361003 People’s Republic of China; 2 Department of Medical Oncology, People’s Hospital of Baise, Baise, 533000 People’s Republic of China; 3 Department of Obstetrics and Gynecology, The First Affiliated Hospital of Xiamen University, Xiamen, 361003 People’s Republic of China; 4 Department of Oncology, Dongguan Third People’s Hospital, Affiliated Dongguan Shilong People’s Hospital of Southern Medical University, Dongguan, 523326 People’s Republic of China; 5 Xiamen Key Laboratory of Chiral Drugs, School of Medicine, Xiamen University, Xiamen, 361005 People’s Republic of China

**Keywords:** Ovarian cancer, Circ-PTK2, miR-639, FOXC1, Epithelial–mesenchymal transition

## Abstract

**Background:**

Precise quantification of microRNA is challenging since circulating mRNA and rRNA in the blood are usually degraded. Therefore, it is necessary to identify specific biomarkers for ovarian cancer. This study aimed to investigate candidate circular RNAs (circRNAs) involved in the pathogenic process of ovarian cancer after inhibition of chromodomain helicase DNA binding protein 1-like (CHD1L) and the corresponding mechanism.

**Methods:**

CHD1L mRNA-targeted siRNA was designed and induced a decreased level of CHD1L function in SK-OV-3 and OVCAR-3 cells observed via transwell and wound healing assays and assessment of epithelial–mesenchymal transition (EMT)-related protein expression by immunofluorescence (IF) and western blotting (WB). After decreasing the level of CHD1L, RNA-seq was conducted, and the circRNA expression profiles were obtained. cirRNAs were then selected and validated by PCR together with Sanger sequencing, fluorescent in situ hybridization (FISH), and reverse transcriptase-quantitative PCR (RT-qPCR). Selected circRNA function in vitro was adjusted via interference and overexpression and assessed via transwell assay, tube formation, and EMT-related protein assay by IF and WB; tumor formation in vivo was followed via hematoxylin and eosin (HE) staining and immunohistochemistry of EMT-related proteins. Based on the competing endogenous RNA prediction of circRNA targets, candidate miRNAs were found, and their downstream mRNAs targeted by the selected miRNA were identified and validated by luciferase assay. The functions of these selected miRNA and mRNA were then further investigated through transwell and WB assay of EMT-related proteins.

**Results:**

CHD1L was significantly upregulated in ovarian cancer tissues and patients with higher expression of CHD1L had a shorter relapse-free survival (*P* < 0.001) and overall survival (*P* < 0.001). Inhibiting the level of CHD1L significantly decreased cell migration and invasion (P < 0.05), increased the expression of epithelial markers, and decreased the expression of mesenchymal markers. Following inhibition of CHD1L expression, RNA-seq was conducted and 82 circRNAs had significantly upregulated expression, while 247 had significantly downregulated expression. The circRNAs were validated by PCR, and hsa_circ_0008305 (circ-PTK2) was selected and further validated by Sanger sequencing, FISH, and RT-qPCR. Circ-PTK2 expression was significantly higher in the ovarian cancer tissues compared with normal ovary tissues (*P* < 0.001). By regulating the level of circ-PTK2 with siRNA and an overexpression vector, expression of circ-PTK2 was found to be positively correlated to cell migration and invasion. Overexpression of circ-PTK2 enhanced tumor formation and was correlated to expression of EMT pathway markers. Prediction of the target of circ-PTK2 was validated with dual luciferase assay and identified miR-639 and FOXC1 as the valid target of circ-PTK2 and miR-639, respectively. The RNA level of miR-639 was negatively correlated to cell proliferation and migration, whereas the mRNA level of FOXC1 was positively correlated to those processes. miR-639 mimics reversed the function of circ-PTK2 overexpression; however, interference of FOXC1 mRNA also reversed the function of circ-PTK2.

**Conclusions:**

circ-PTK2 is an important molecule in regulating the pathogenic processes of ovarian cancer via the miR-639 and FOXC1 regulatory cascade.

**Supplementary Information:**

The online version contains supplementary material available at 10.1186/s12935-021-01985-x.

## Background

Ovarian cancer is a malignancy with a 45% 5-year survival rate that significantly affects patient health globally [1]. Following improvements and developments in the diagnosis and treatment of this disease, the overall survival of patients has been controlled. Based on current studies, 70% of patients are diagnosed at a late stage of ovarian cancer and consequently only have a 25% survival rate, whereas the 5-year survival rate is higher for those diagnosed at an earlier stage [2, 3]. Specific biomarkers can be used to screen for the disease and therefore limit the high rate of ovarian cancer recurrence [4]. CA125 is a common diagnostic biomarker used for ovarian cancer screening [5] but has poor specificity and can only help to screen half of the patients at early disease stages and 70–90% at later stages [6]. Another biomarker, human epididymis protein 4 (HE4), presents similar sensitivity and specificity for ovarian cancer compared with CA125. However, both biomarkers show contradicting accuracy of diagnosis [7]. Other biomarkers such as microRNA have also been considered as biomarkers and have potential clinical utility for prognosis prediction in ovarian cancer. However, precise quantification of microRNA is challenging since circulating mRNA and rRNA in the blood are usually degraded [8]. Therefore, it is necessary to identify specific biomarkers for ovarian cancer.

Circular RNAs (circRNAs) are a class of noncoding RNAs reported as indicators in several cellular activities [9–11]. circRNAs can be generated by backsplicing of introns and exons and are expressed in the cytoplasm, where the circRNA loop structure prevents degradation from RNA exonucleases [10]. Several reports have indicated that circRNAs might have important regulatory roles in specific processes. Evidence has suggested that circRNAs act as miRNA sponges, which perform functions by interacting with miRNAs [12]. The expression profiles of circRNAs also showed that circRNAs are highly specific for certain tissues and pathological conditions [13, 14]. Therefore, circRNAs can potentially be used as disease biomarkers and therapeutic targets. Recently, studies have indicated that circRNAs might participate in the development and progression of cancer [15–17]. Salzman et al. reported that expression of circRNAs in cancerous and non-cancerous cell lines and tissues correlated to the presence of acute lymphoblastic leukemia [14]. Dysregulation of circRNA has been found in several human cancers, such as bladder cancer, breast cancer, hepatocellular carcinoma, gastric cancer, and prostate adenocarcinoma [18]. In addition, circ-001567 has been reported to promote ovarian cancer cell proliferation and invasion [19].

In this study, we attempted to suppress chromodomain helicase DNA binding protein 1-like (CHD1L) expression, which is reported to be highly expressed in ovarian carcinoma and correlated to metastasis [20], and screen potential circRNAs by sequencing. Furthermore, cell assays were conducted to demonstrate the functions of the circRNAs in carcinogenesis leading to ovarian cancer. The corresponding regulatory pathways and miRNAs and mRNAs were predicted and validated. This understanding of circRNAs may enable their use for diagnosis and treatment of ovarian cancer.

## Materials and methods

### Human samples

Twenty-six ovarian cancer tissue and 11 normal ovary tissue were obtained at the Department of Obstetrics and Gynecology, the First Affiliated Hospital of Xiamen University (Xiamen, China). No patients receiving chemotherapy or radiotherapy before surgery. All the enrolled patients provided informed consents. Our study was approved by the Ethics Committee of the First Affiliated Hospital of Xiamen University.

### Reverse transcriptase-quantitative PCR (RT-qPCR)

RNA was isolated using TRIzol reagent (ThermoFisher). cDNA was reversed transcribed using M-MLV reverse transcriptase (ThermoFisher). qPCR was conducted using GoTaq qPCR kit (Promega, Madison, WI, USA). U6 was used as internal reference for miRNA expression, whereas GAPDH was used as an internal reference for circRNA and mRNA expression. Specific primers are shown in Table 1.

**Table 1:** Specific primers sequence

Primer name	Primer sequence (5ʹ-3ʹ)
hsa_circ_0005265 divergent-F	ACGGATGCCAGAACAGAACC
hsa_circ_0005265 divergent-R	GTTGCCAGTGAGAGAAATCAGC
hsa_circ_0005265 convergent-F	GGCCAAAACTTCAAATCCAA
hsa_circ_0005265 convergent-R	GCTGTTCTTGTGGTCCCATT
hsa_circ_0008305 divergent-F	CGTCTCTGTGTCAGAAAAGATGT
hsa_circ_0008305 divergent-R	AGGTTGGCAAATTGTCTAAATGT
hsa_circ_0008305 convergent-F	GGATTCTGTCAAGGCCAAAA
hsa_circ_0008305 convergent-R	CAGCTTGAACCAAGAGCACA
hsa_circ_0003171 divergent-F	CCTCAGCTAGTGACGTATGGA
hsa_circ_0003171 divergent-R	ACTGACGCATTGTTAAGGCT
hsa_circ_0003171 convergent-F	TGTACTTCGGACAGCGTGAG
hsa_circ_0003171 convergent-R	GTGTGCACAGCTCCATGATT
hsa_circ_0005990 divergent-F	AGCAGGATGGTGGGACTCAA
hsa_circ_0005990 divergent-R	TCTGGTTCATGGCTGTTAAGGA
hsa_circ_0005990 convergent-F	CAGCAACTGCAGATGGAGAA
hsa_circ_0005990 convergent-R	TGGATTTTGAGTCCCACCAT
hsa_circ_0037002 divergent-F	CAGAAGCCTCATTTGCCTGC
hsa_circ_0037002 divergent-R	CTTTAATTTGGCCTGCATTACTGA
hsa_circ_0037002 convergent-F	AAGTGAACCTGTCCCCATTG
hsa_circ_0037002 convergent-R	TTGTCAGGTTTGTCGAGCTG
GAPDH convergent-F	GAGTCAACGGATTTGGTCGT
GAPDH convergent-R	GAGTCAACGGATTTGGTCGT
GAPDH divergent-F	TCCTCACAGTTGCCATGTAGACCC
GAPDH divergent-R	TGCGGGCTCAATTTATAGAAACCGGG
Chd1l-F	GACCTGAGTTTGGGTGATG
Chd1l-R	CGGATAAGTCGTTCGGTA
E-cadherin-F	GAGAAACAGGATGGCTGAAGG
E-cadherin-R	TGAGGATGGTGTAAGCGATGG
N-cadherin-F	ATGAAAGACCCATCCACGC
N-cadherin-R	CCTGCTCACCACCACTAC
Snail-F	ACCACTATGCCGCGCTCTT
Snail-R	GGTCGTAGGGCTGCTGGAA
Vimentin-F	AGTCCACTGAGTACCGGAGAC
Vimentin-R	CATTTCACGCATCTGGCGTTC
FOXC1-F	TCGGCTTGAACAACTCTCCAG
FOXC1-R	ACAGTCGTAGACGAAAGCTCC
hsa-miR-639-RT	GTCGTATCCAGTGCAGGGTCCGAGGTATTCGCACTGGATACGACACAGCG
hsa-miR-639-F	GCCGAGATCGCTGCGGTTGCGAG
Universe R	GTGCAGGGTCCGAGGT
hsa-U6-F	CTCGCTTCGGCAGCACA
hsa-U6-R	AACGCTTCACGAATTTGCGT

### Cell culture

SK-OV-3 and OVCAR-3 cell lines were purchased from Cellcook Biotech Co., Ltd (Guangzhou, China). SK-OV-3 cells were cultured in McCoy’s 5A media (Sigma-Aldrich, St. Louis, MO, USA), adding NaHCO_3_ to 2.2 g/L, with 10% fetal bovine serum (FBS, Gibco, Grand Island, NY, USA). OVCAR-3 cells were cultured in RPMI 1640 (Gibco) with 20% FBS and 10 μg/mL insulin (Cellcook). Both cell lines were cultured in 5% carbon dioxide at 37 ℃.

### Cell transfection

siRNAs targeted to CHD1L mRNA and circ-PTK were synthesized by GenePharma (Shanghai, China). The specific interference fragment sequences are shown in Table 2. For transfection, cells were seeded in 6-well plates and incubated in medium. When the cell density reached 80% confluence, medium was removed and refreshed with 2 mL complete growth medium with 10% FBS (ThermoFisher, USA). siRNA (200 pmol) dissolved in 150 μL opti-MEM (GIBCO) was mixed with EndoFectin-Max (GeneCopoeia, USA) transfection reagent diluted in 150 μL opti-MEM; the mix was incubated at room temperature for 20 min and then added to the 6-well plates. Cells were then incubated as above and collected at the designated time for subsequent detection.

**Table 2:** The specific interference fragment sequence

Name	Sequence (5ʹ-3ʹ)
CHD1L-1 sense	UAUUGGACAUGCCACGAAA
CHD1L-1 anUisense	UUUCGUGGCAUGUCCAAUA
CHD1L-2 sense	GGAGACUCAUAGAGGAGAA
CHD1L-2 anUisense	UUCUCCUCUAUGAGUCUCC
circ-PUK2 sense	GUCAGAAAAGAUGUUGGUUUA
circ-PUK2 anUisense	UAAACCAACAUCUUUUCUGAC
si-NC sense	UUCUCCGAACGUGUCACGU
si-NC anUisense	ACGUGACACGUUCGGAGAA
miR-639 mimics sense	AUCGCUGCGGUUGCGAGCGCUGU
miR-639 mimics anUisense	ACAGCGCUCGCAACCGCAGCGAU
miR-639 inhibiUor	ACAGCGCUCGCAACCGCAGCGAU
inhibiUor NC	CAGUACUUUUGUGUAGUACAA

### Transwell assay to detect cell migration and invasion

Cells were digested and adjusted to 1 × 10^6^ cells/mL in culture medium without serum. For the cell migration assay, 100 μL of cell suspension was added to the upper chamber, and 600 μL of cell culture medium with FBS was added to the bottom chamber. For the cell invasion assay, Matrigel (BD Biosciences, San Jose, CA, USA) was added to the upper chamber and incubated at 37 ℃ for 2 h. Then, 100 μL and 600 μL of culture medium was added to the upper and bottom chambers, respectively, and incubation continued at 37 ℃ overnight. The culture medium was removed and 100 μL of cell suspension was added to the upper chamber, and 600 μL of cell culture medium with FBS was added to the bottom chamber.

For both assays, cells were incubated at 37 ℃ and 5% CO_2_ environment for 24 h. The upper chamber was isolated, and the cells were removed and fixed with 4% paraformaldehyde. Cells were washed with phosphate buffer saline (PBS) and stained with crystal violet. The stained cells were observed by microscopy (Olympus, Tokyo, Japan).

### Wound healing assay

SK-OV-3 and OVCAR-3 cells were seeded to 6-well plates at 1 × 10^6^ cells per well. After cells had adhered, 1 μg/mL of mitomycin C was added to the cells to inhibit cell proliferation. A 200 μL tip was used to generate a wound on the cell layer. Cells were washed by PBS to remove loose cells from the plate and cell culture medium added to the wells. The cells were cultured at 37 ℃ and photographed at 0, 12, 24, and 48 h.

### Tube formation assay

SK-OV-3 and OVCAR-3 cells were transfected with siRNA and cultured for 48 h. Cells were then washed twice with PBS and refreshed with non-serum culture medium. After 24 h of incubation, the supernatant was collected for the tube formation assay. HUVEC cells were seeded and cultured with the HUVEC culture medium mixed with the collected supernatant at 1:1 ratio. Matrigel (100 μL) was added to a 48-well plate. After the gel had set, 50 μL of HUVEC cell suspension was added at 8 × 10^5^ cell/mL. The HUVEC cells were incubated at 37 ℃ and photographed at 6 h. The amounts and length of the notches were analyzed by ImageJ software 1.8 (National Institutes of Health, Bethesda, MD, USA).

### Immunofluorescent (IF) staining

Cells were fixated with 4% Paraformaldehyde and incubated at 4 ℃ for 10 min. PFA was removed, and cells were permeabilized with 0.1% Tyrion X-100 for 5 min. Cells were blocked with 3% FBS (Gibco) for 1 h. Cells were then washed by PBS three times and incubated with primary antibody (E-cadherin, CST 14472; N-cadherin, CST 4061P). After incubating for 1 h in the dark, cells were washed by PBS three times and incubated with secondary antibody (Alexa Flour 488 Don anti-Ms IgG, H+L: A21202, Invitrogen) for 1 h in the dark. Cells were washed by PBS three times and stained with 1× Hoechst (Invitrogen) for 10 min in the dark. Cells were washed by PBS three times and blocked by mounting medium.

### Western blotting

Cells were lysed with RIPA reagent (Beyotime Biotechnology, Shanghai, China). The lysate was centrifuged, and supernatant collected. The protein was quantified by BCA method and then separated on 8% SDS-PAGE followed by transfer to PVDF membrane. The membrane was blocked by non-fat milk and incubated with primary antibody, E-cadherin (CST, 14472; 1:1000), N-cadherin (CST, 4061P; 1:1000), vimentin (Santa, 6260; 1:1000), or GAPDH (Proteintech, 60004-1-lg; 1:8000). The membrane was washed twice by Tris-Buffered Saline and Tween 20 and incubated with secondary antibody for 2 h (Forevergen; 1:1000). The membrane was incubated with ECL (Forevergen, Guangzhou, China) after washing with TBST, and the bands observed.

### RNA-seq

Total RNA from the cells was purified using RNeasy Mini Kit (QIAGEN, Dusseldorf, Germany). RNA integrity was evaluated based on the RIN value using Agilent bioanalyzer 2100 (Agilent, CA, USA). RNA was cleaned with the RNA Clean XP Kit (Beckman Coulter, CA, UA), and the DNA residue was removed by RNase-free DNase Set (Qiagen). The quality and concentration of the RNA were determined by NanoDrop 2000 (ThermoFisher). The rRNA was removed by NEBNext rRNA Depletion Kit (NEB, USA). Total RNA (1 μg) was used for library preparation using the VAHTSTM mRNA-seq v2 library Prep Kit (Vazyme, Nanjing, China). The RNA was fragmented, and cDNA was synthesized. End polishing was performed and the cDNA fragments were ligated with adapters. The ligated cDNA was amplified with universal PCR primers to obtain sufficient library for sequencing. Agilent bioanalyzer 2100 (Agilent, Santa Clara, CA, USA) was used to evaluate the quality of the library. Illumina Hiseq 4000 (Illumina) was used for the RNA sequencing. The data was then assembled and annotated with corresponding symbol of transcripts. The differentially expressed circRNAs were screened using R software according to P-value ≤ 0.05, |log2Ratio|≥ 1.

### Luciferase reporter assay

The circ-PTK2 wild-type (WT) sequence and mutated (Mut) sequence and the FOXC1 WT sequence and Mut sequence were cloned into PmirGLO vector, synthesized by General Biol (Anhui, China). miR-639 mimics and miRNA negative control were synthesized by GenePharma. The reporter and miR-639 mimics and miRNA negative control were transfected to 293T cells using Lipofectamine 2000 transfection reagent (Thermo Fisher), and dual luciferase reporter assays conducted (Promega).

### Fluorescent in situ hybridization (FISH)

The circ-PTK2 probe (5′-CTTTAAACCAACATCTTTTC TGACACAGAGACGGCGTGT-3′) was provided by GenePharma. FISH was conducted (GenePharma), and probe signal on cells was observed under fluorescent microscopy (Carl Zeiss, Jena, Germany).

### Tumor formation assay

Female nude 4–5-week-old mice were used for the assay. After transfection of circ-PTK2 plasmids or control plasmids for 48 h, SK-OV-3 cells (5 × 10^6^ in 100 μL) were injected to the abdomen. The mice were euthanized and the number of nodules was counted. The volume of the tumor was calculated following (L × W2)/2. The tumor was further assessed with hematoxylin and eosin (HE) staining and immunohistochemistry. All experiments and procedures were reviewed and approved by the Ethics Committee of the First Affiliated Hospital of Sun Yat-sen University (Guangzhou, China).

### HE staining

HE staining according to the Intruduction of kit (Beyotime Biotechnology, Shanghai, China).

### Immunohistochemical (IHC) staining

The slides were deparaffinized by incubating in xylene for 15 min and were then washed with 100% ethanol for 5 min, 95% ethanol for 5 min, and 75% ethanol for 5 min. The slides were washed with water followed by 0.01 M sodium citrate buffer (pH 6.0) and 100 ℃ for 3 min; the slides were then cooled at room temperature for 30 min and washed with water. The slides were blocked with 3% H_2_O_2_ for 15 min and washed by PBS three times; slides were then blocked with 5% BSA for 15 min at room temperature. Primary antibody was added, and the slides were incubated at 4 ℃ overnight; the slides were washed with PBS three times. Secondary IgG antibody with HRP label was added, and the slides were incubated at room temperature for 1 h; the slides were washed by PBS three times. DAB was added and incubated for 10 min to develop color. The reaction was stopped by washing the slides with water. The slides were stained with hematoxylin for 1 min, washed by water and 1% hydrochloric acid alcohol, and rinsed with water until they turned blue. The slides were incubated in 75%, 95%, and finally 100% ethanol. The slides were then incubated in 100% xylene for 10 min and then blocked. The slides were observed and photographed by microscopy. The primary antibodies used were E-cadherin (CST, 14472, 1:1000) and N-cadherin (CST, 4061P, 1:1000).

### Statistical analysis

Normally distributed data were compared using independent t-tests, one-way and two-way ANOVA. ALL data are presented as mean ± standard deviation (SD) of three independent experiments. A *P*-value < 0.05 was taken as indicating significant difference. SPSS 22.0 software (SPSS Inc, Chicago) and GraphPad Prism, version 8.0 (GraphPad Software) were used for the statistical analysis.

## Results

### CHD1L is highly expressed in ovarian cancer and affects patient survival

**Fig. 1:**
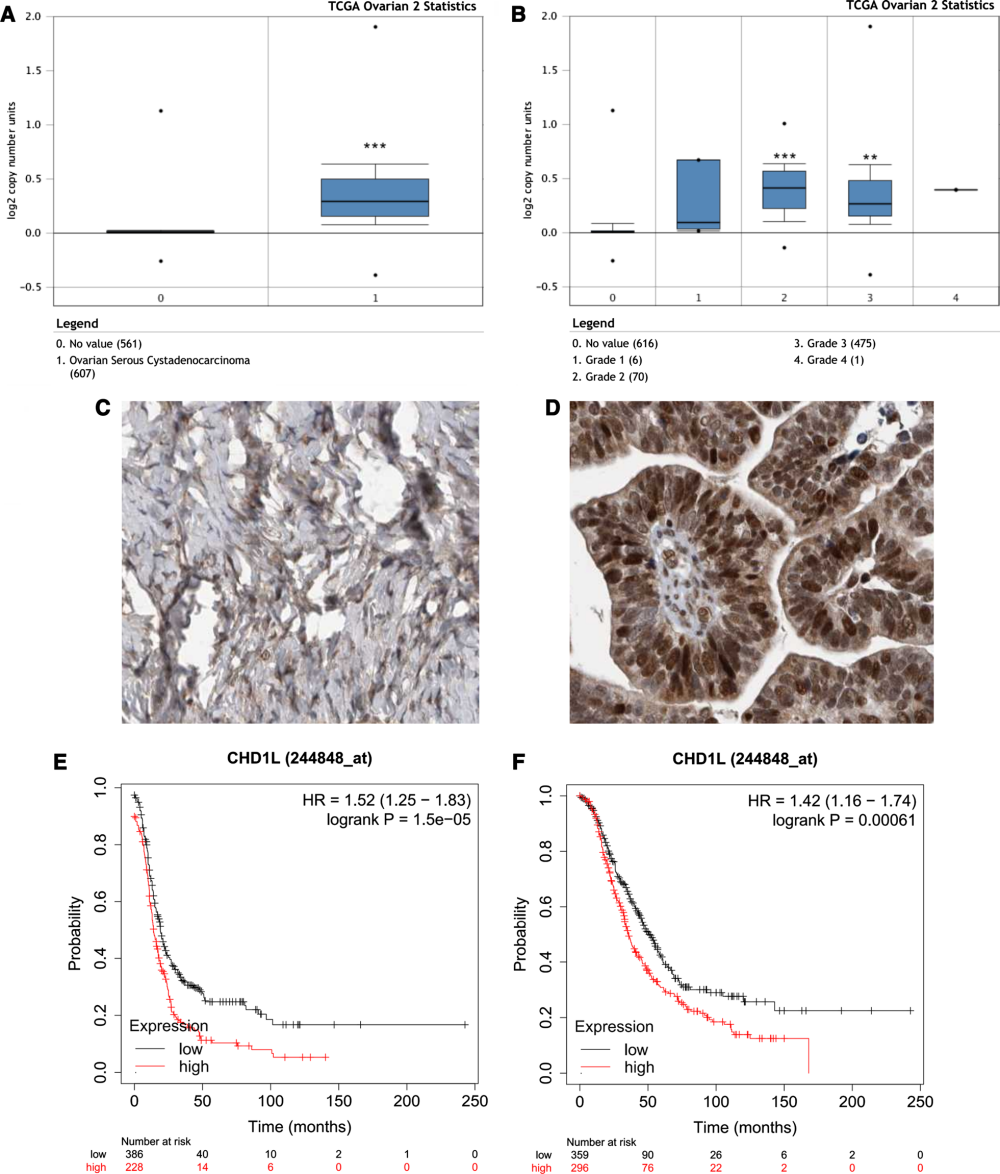
CHD1L is highly expressed in ovarian cancer tissues and affects patient survival. **A** CHD1L mRNA expression in ovarian serous cystadenocarcinoma was significantly higher than normal ovarian tissue (Oncomine analysis). **B** CHD1L mRNA expression in grade II and grade III ovarian serous cystadenocarcinoma were significantly higher than normal ovarian tissue or grade I ovarian serous cystadenocarcinoma (Oncomine analysis). **C**, **D** The protein expression of CHD1L in ovarian serous cystadenocarcinoma (**D**) was higher than normal ovarian tissue (**C**) (Human Protein Atlas analysis). **E** Increased CHD1L expression was related to an inferior relapse-free survival in ovarian cancer patients. **F** Increased CHD1L expression was related to an inferior overall survival in ovarian cancer patients

In our previous study, we have found that the expression of CHD1L was significantly higher in metastatic lesions of ovarian cancer compared to the primary lesions (*P* < 0.05) [20]. In addition, the level of CHD1L expression was significantly higher in serous subtype compared to mucinous subtype (*P* = 0.029) [20]. Moreover, higher expression of CHD1L was significantly associated with inferior patient survival [20]. In the present study, to evaluate changes in CHD1L expression in ovarian cancer tissues and normal ovarian tissues, we analyzed the transcriptional levels of CHD1L through several independent bioinformatics databases. Using the Oncomine database (http://www.oncomine.org) [21], we also found that CHD1L expression was significantly increased in ovarian serous cystadenocarcinoma compared to normal ovarian tissue (*P* < 0.001) (Fig. 1A). After stratification by tumor grade, CHD1L mRNA expression in grade II and grade III ovarian serous cystadenocarcinoma were significantly higher than normal ovarian tissue or grade I ovarian serous cystadenocarcinoma (Fig. 1B). Using the database of Human Protein Atlas (https://www.proteinatlas.org), the protein of CHD1L expression was also significantly increased in ovarian serous cystadenocarcinoma compared to normal ovarian tissue (Fig. 1C, D). Using the KM plotter (http://kmplot.com/analysis/) [22], we found that patients with higher expression of CHD1L had a shorter relapse-free survival (RFS) (*P* < 0.001) (Fig. 1E) and overall survival (OS) (*P* < 0.001) (Fig. 1F) than those with lower expression of CHD1L. In conclusion, CHD1L was significantly upregulated in ovarian cancer tissues and impacted the survival of ovarian cancer patients, suggesting its oncogenic role.

### CHD1L regulates cell migration and invasion

**Fig. 2:**
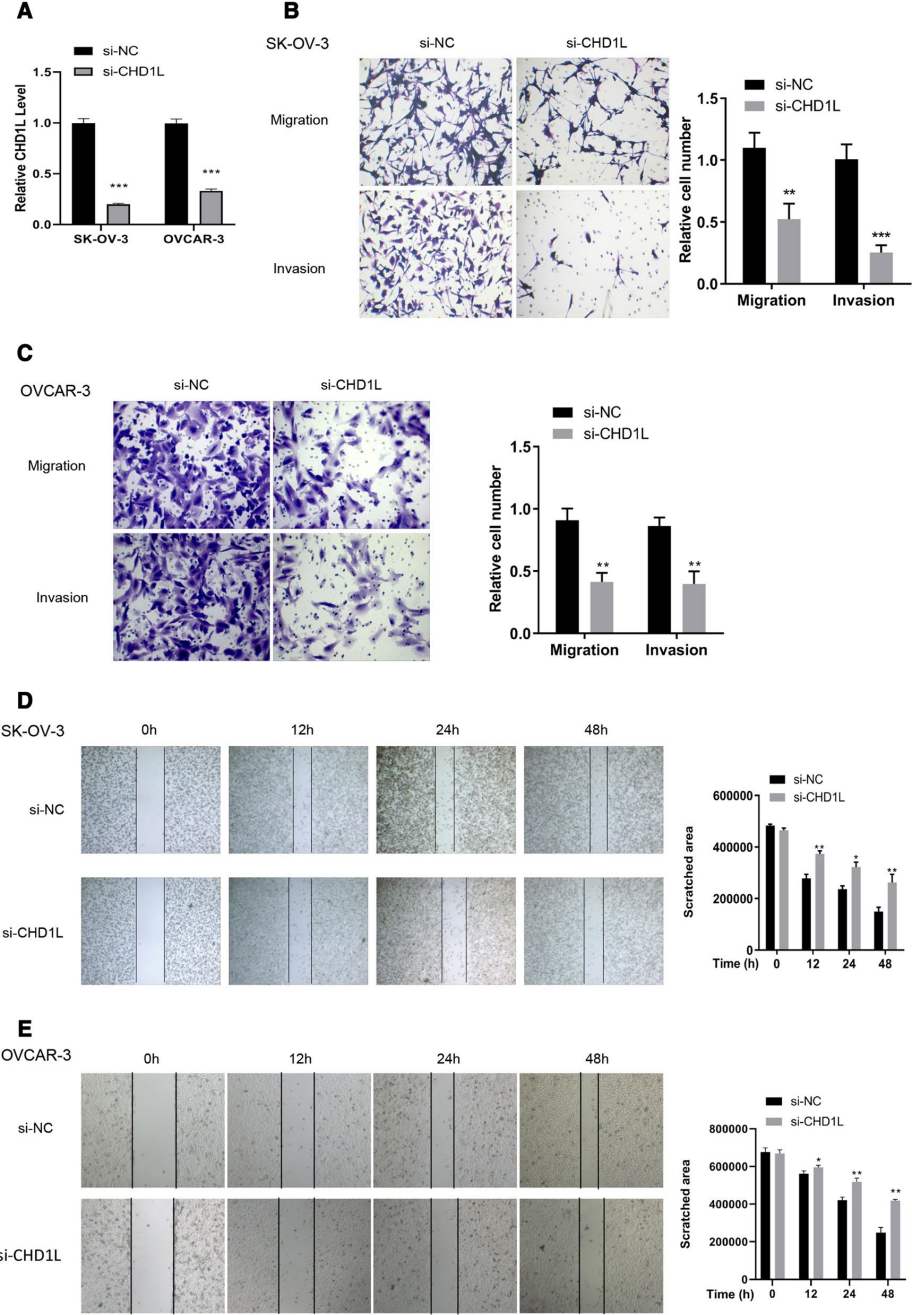
CHD1L regulates the cell migration and invasion in SK-OV-3 and OVCAR-3 cells. **A** The relative level of CHD1L was significantly decreased under the effect of si-CHD1L in SK-OV-3 and OVCAR-3 cells. **B** Cell migration and invasion was suppressed when the level of *CHD1L* mRNA was decreased by si-CHD1L in SK-OV-3 cells. **C** Cell migration and invasion was suppressed when the level of *CHD1L* mRNA was decreased by si-CHD1L in OVCAR-3 cells. **D** Cell migration affected by inhibition of CHD1L expression in SK-OV-3 cells validated by wound healing assay. **E** Cell migration affected by inhibition of CHD1L expression in OVCAR-3 cells validated by wound healing assay. Data are presented as the mean ± SD. *P < 0.05; **P < 0.01; ***P < 0.001

To validate the functions of CHD1L in ovarian cancer, a specific siRNA was designed and transfected into SK-OV-3 and OVCAR-3 cells. si-CHDIL treatment decreased the relative CHD1L level to 20% in SK-OV-3 cells (*P* < 0.001) and to 40% in OVCAR-3 cells (*P* < 0.001) compared with that of control group, transfected with siRNA negative control (si-NC) (Fig. 2A). This indicated that the siRNA has been successfully transfected into the cells and that significantly downregulated the expression of CHD1L mRNA. SK-OV-3 and OVCAR-3 cells transfected with si-CHD1L and si-NC were tested in transwell assays to determine the capacity of cell migration and invasion. Cell numbers for migrating and invasive cells decreased < 50% for both the transfected SK-OV-3 (Fig. 2B) and OVCAR-3 (Fig. 2C) cell lines than those in the si-NC group. After 12 h, there was a significant difference in the size of the scratched area in both the si-CHD1L transfected cell lines than that in the si-NC group (Fig. 2D, E), indicating that the cell migration capacity was suppressed when the expression of CHD1L was inhibited.

**Fig. 3:**
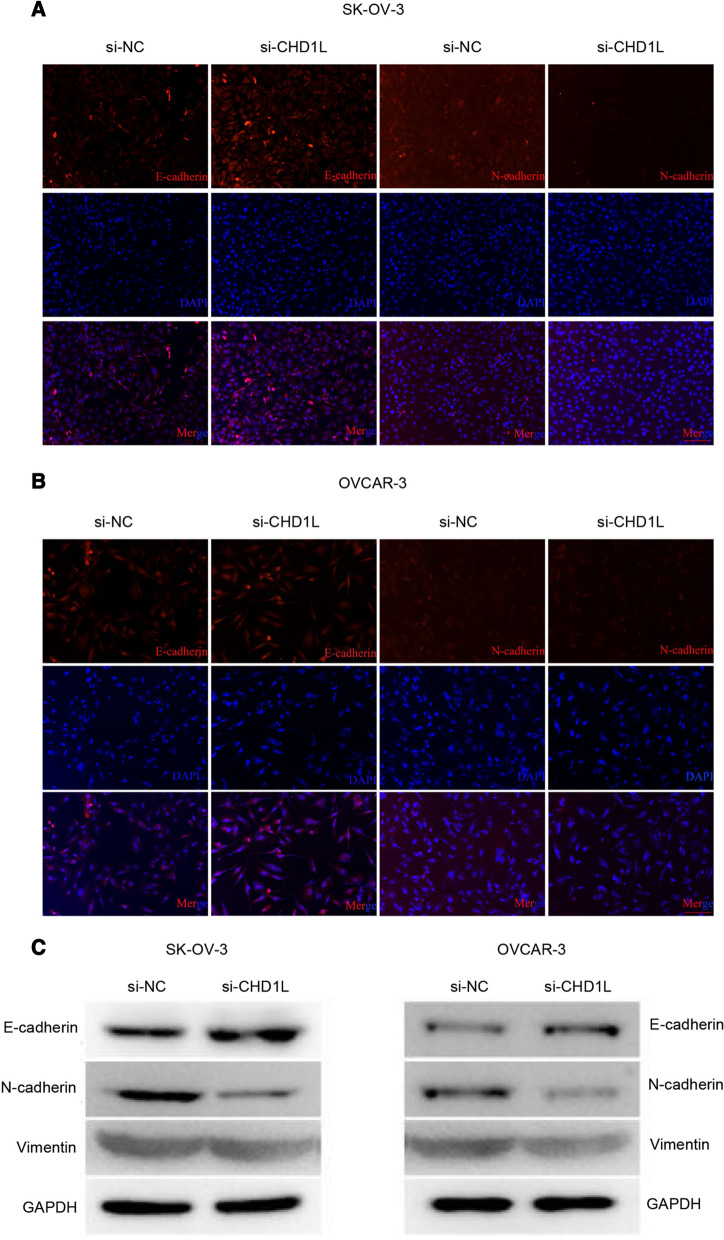
The level of CHD1L correlates to levels of proteins in the epithelial–mesenchymal transition (EMT) pathway. **A** Fluorescent in situ hybridization (FISH) to determine the protein levels of E-cadherin and N-cadherin in SK-OV-3 cells. **B** FISH to determine the protein levels of E-cadherin and N-cadherin in OVCAR-3 cells. **C** Western blotting to determine the protein levels of the EMT pathway-related genes, E-cadherin, N-cadherin, and vimentin in SK-OV-3 and OVCAR-3 cells

FISH was conducted to validate whether the cell migration and invasion correlated to expression of markers in the epithelial–mesenchymal transition (EMT) pathway. The majority of si-CHDIL-transfected SK-OV-3 and OVCAR-3 cells expressed E-cadherin, but fewer cells expressed N-cadherin, compared with those of the si-NC group (Fig. 3A, B). WB was performed to further validate the protein level of the corresponding EMT genes. This demonstrated that inhibition of CHD1L mRNA expression by siRNA increased the protein level of E-cadherin but decreased the protein level of N-cadherin and vimentin in both SK-OV-3 and OVCAR-3 cells, indicating that the EMT pathway was suppressed. Therefore, we showed that the expression of CHD1L is positively correlated to ovarian cell migration and invasion.

### Candidate circRNAs involves in the regulation of CHD1L-related functions

**Fig. 4:**
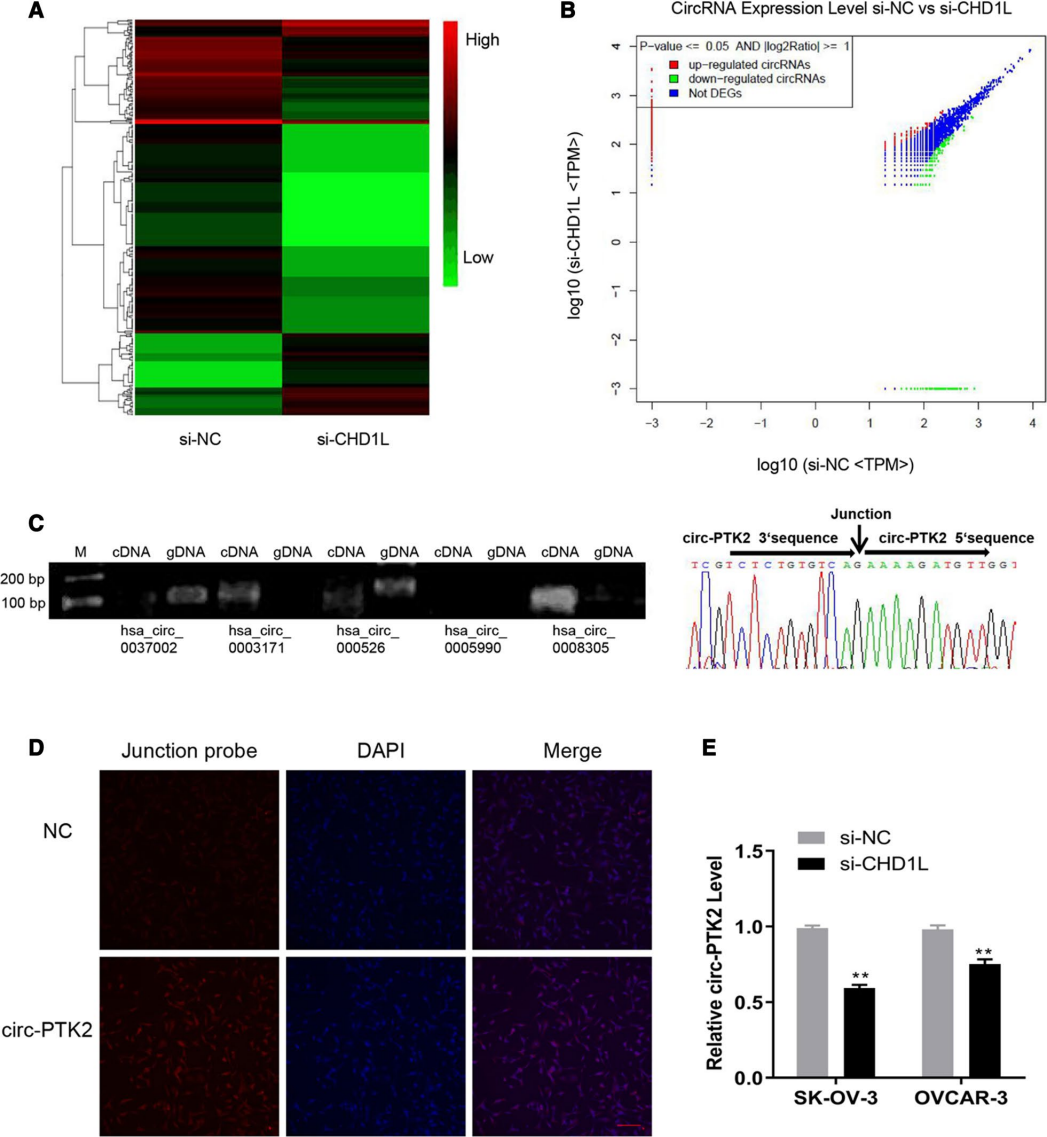
Circ-PTK2 was identified as the candidate circRNA involved in ovarian cancer after CHD1L was inhibited. **A** Cluster graph in presenting the expression profiles of SK-OV-3 cells with si-CHD1L or si-NC treatment. **B** Dot plots of differentially expressed circRNAs, with upregulated expression of 82 circRNAs and downregulated expression of 247 circRNA. **C** Divergent and convergent primers for PCR and Sanger sequencing to validate the circular structure of the candidate circular RNAs. **D** Fluorescent in situ hybridization validation of the expression level of circ-PTK2. **E** Reverse transcriptase-quantitative PCR validation of the level of circ-PTK2 in SK-OV-3 and OVCAR-3 cells after inhibition of CHD1L expression. Data are presented as the mean ± SD. **P < 0.01

The total RNA from SK-OV-3 cells transfected with siCHD1L or si-NC was purified and then analyzed by RNA-seq to verify the circRNAs involved in the regulation; the expression profiles were summarized in the cluster plot (Fig. 4A) and dot plot (Fig. 4B). Based on the analysis, there was upregulated expression of 82 circRNAs and downregulated expression of 247 circRNAs in the siCHD1L-treated group than those in the si-NC-treated group. Among these circRNAs, we chose those that correlated to the cancerous process and were highly expressed in PCR validation using divergent and convergent primers on cDNA and gDNA. circRNAs can be amplified from cDNA but cannot be amplified from gDNA due to their circular structure. The candidate circRNAs that reached this criteria included hsa_circ_0003171 and hsa_circ_0008305 (also known as circ-PTK2) (Fig. 4C). We chose circ-PTK2 for further study as the expression of this circRNA was higher than that of hsa_circ_0003171. The circular structure was validated using Sanger sequencing, which showed that the junction sequences were clearly detected from products of the hsa_circ_0008305 convergent primer (Fig. 4C). We then designed a probe based on the junction sequences of circ-PTK2 and conducted FISH. This demonstrated that circ-PTK2 was expressed in the cells and that signal was detected in both the cytoplasm and nucleus, indicating that circ-PTK2 can perform functions in both locations (Fig. 4D).

To validate the correlation between CHD1L and circ-PTK2, the RNA level of circ-PTK2 was determined in the SK-OV-3 and OVCAR-3 cells after inhibition of CHD1L expression. The RNA level of circ-PTK2 was significantly decreased when CHD1L was inhibited in SK-OV-3 (*P* < 0.01) and OVCAR-3 cells (*P* < 0.01) (Fig. 4E), indicating that the expression of circ-PTK2 positively correlated with that of CHD1L.

### Correlation of circ-PTK2 expression with ovarian cancer

**Fig. 5:**
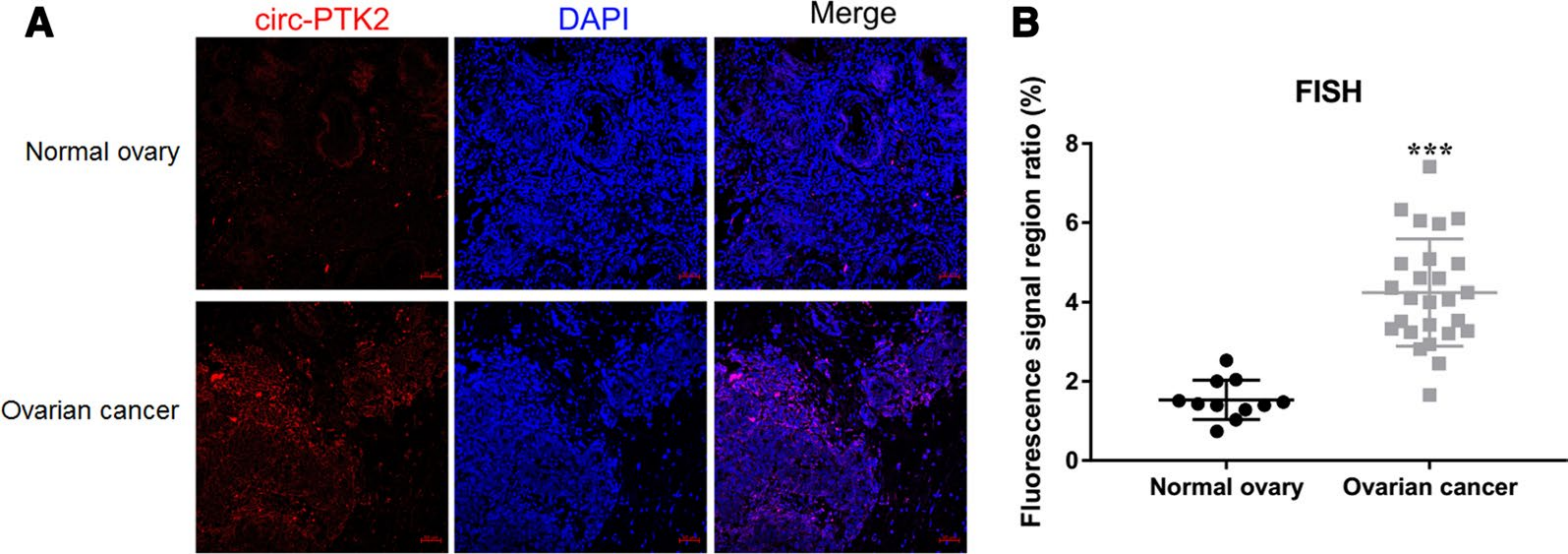
Correlation of circ-PTK2 expression with ovarian cancer. **A** The location of circ-PTK2 in ovarian cancer tissues was detected using FISH assay. **B** Differentially expressed circ-PTK2 in ovarian cancer tissues and normal ovary tissues were measured by FISH. Data are presented as the mean ± SD. ***P < 0.001

We collected 26 ovarian cancer samples and 11 normal ovary samples to analyze the level of circ-PTK2 between ovarian cancer and normal ovary samples using FISH. Circ-PTK2 was specifically expressed in the cytoplasm of the ovarian cancer tissue (Fig. 5A). Circ-PTK2 expression was significantly higher in the ovarian cancer tissues compared with normal ovary tissues (*P* < 0.001) (Fig. 5B).

### Circ-PTK2 affects migration, invasion, angiogenesis, and EMT process of ovarian cancer

**Fig. 6:**
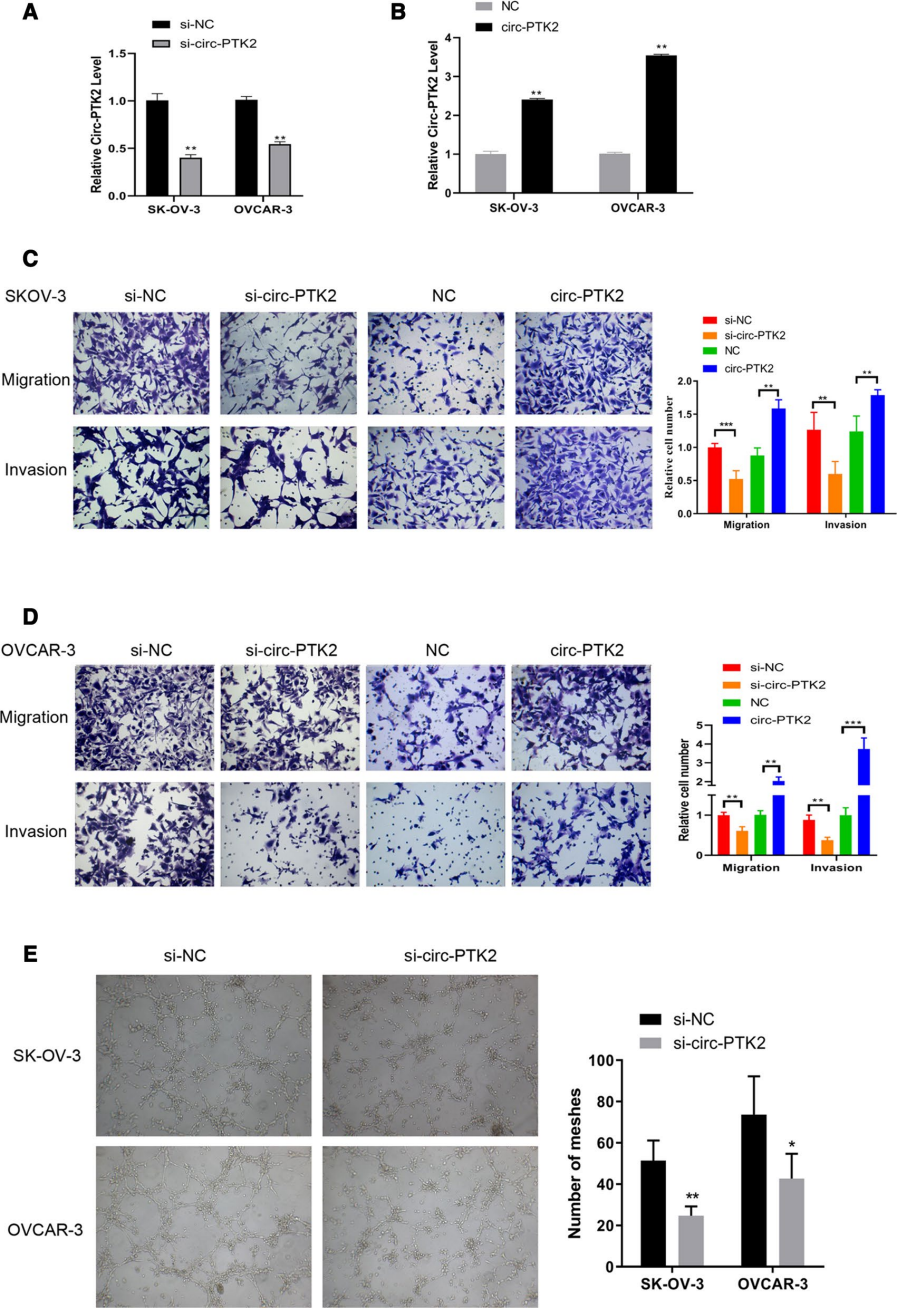
Circ-PTK2 performs functions in regulating cell migration, invasion, and angiogenesis. **A** Validation of the siRNA targeting on circ-PTK2 via reverse transcriptase-quantitative PCR (RT-qPCR) in SK-OV-3 and OVCAR-3 cells. **B** Validation of circ-PTK2 overexpression vector by RT-qPCR in SK-OV-3 and OVCAR-3 cells. **C** Transwell assay showing cell migration and invasion in SK-OV-3 cells after inhibition or overexpression of circ-PTK2. **D** Transwell assay showing cell migration and invasion in OVCAR-3 cells after inhibition or overexpression of circ-PTK2. **E** Angiogenesis was determined by Matrigel assay after inhibition of expression of circ-PTK2. Data are presented as the mean ± SD. *P < 0.05; **P < 0.01; ***P < 0.001

**Fig. 7:**
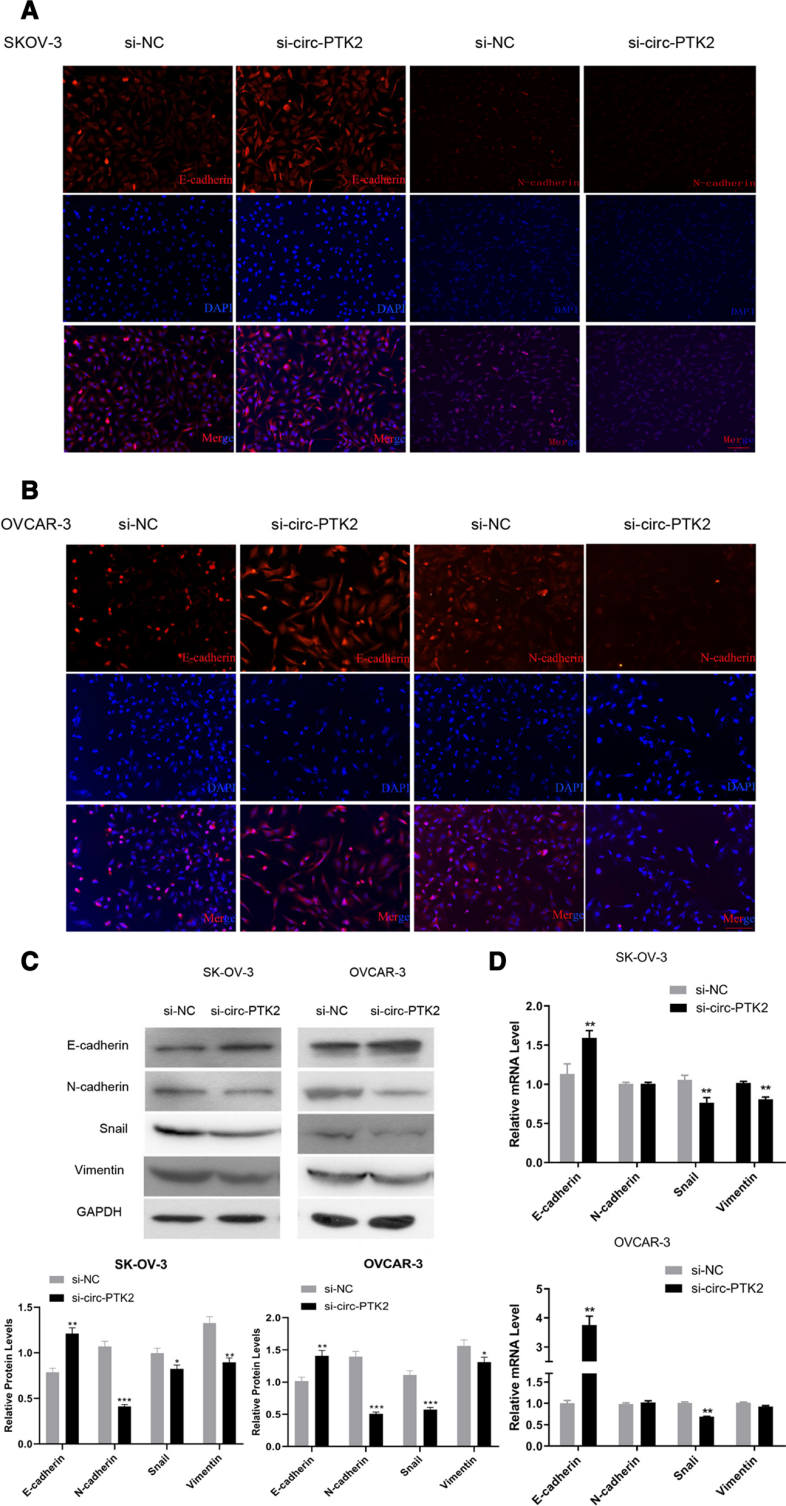
circ-PTK2 regulates the epithelial–mesenchymal transition (EMT) pathway. **A** FISH to determine the protein levels of E-cadherin and N-cadherin in SK-OV-3 cells. **B** FISH to determine the protein levels of E-cadherin and N-cadherin in OVCAR-3 cells. **C** Western blotting analysis of EMT pathway protein expression, including E-cadherin, N-cadherin, Snail, and vimentin. **D** Reverse transcriptase-quantitative PCR analysis of the mRNA level of E-cadherin, N-cadherin, Snail, and vimentin in SK-OV-3 and OVCAR-3 after inhibition of circ-PTK2 expression. Data are presented as the mean ± SD. *P < 0.05; **P < 0.01; ***P < 0.001

To further verify the functions of circ-PTK2, the corresponding siRNA and overexpression vector were designed, transfected into SK-OV-3 and OVCAR-3 cells, and then validated by RT-qPCR. The si-circ-PTK2 inhibited the level of circ-PTK2 to ~ 50% against that of the si-NC group (*P* < 0.01) (Fig. 6A), whereas the overexpression vector increased the level of circ-PTK2 to two- to fourfold higher than that of the negative control group (*P* < 0.01) (Fig. 6B), indicating that both siRNA and vector were designed and synthesized successfully. The cell migration and invasion were then investigated accordingly. Decreasing the level of circ-PTK2 significantly reduced the levels of migrated and invasive cells, whereas increasing the level of circ-PTK2 induced larger amounts of migrated and invasive cells in both SK-OV-3 and OVCAR-3 cell lines (Fig. 6C, D). A tube formation assay was conducted to determine the level of angiogenesis, and the meshes were counted and summarized. The number of meshes decreased to ~ 50% when circ-PTK2 was inhibited by siRNA in SK-OV-3 (*P* < 0.01) and OVCAR-3 cells (*P* < 0.05) cell lines (Fig. 6E). Therefore, circ-PTK2 positively regulated cell migration and invasion and also suppressed angiogenesis. To further validate if circ-PTK2 can regulate the EMT pathway, the level of the relevant proteins, E-cadherin and N-cadherin, were determined by immunofluorescence staining. Compared with cells transfected with si-NC, both SK-OV-3 (Fig. 7A) and OVCAR-3 (Fig. 7B) cell lines transfected with si-circ-PTK2 had increased levels of E-cadherin but decreased levels of N-cadherin. The protein and mRNA levels for E-cadherin, N-cadherin, Snail, and vimentin were determined by WB and qRT-PCR, respectively. Following inhibition by si-circ-PTK2, western blot analysis showed that the level of E-cadherin protein was upregulated in both cell lines (*P* < 0.01). The protein levels of N-cadherin, Snail, and vimentin were decreased in both cell lines (Fig. 7C). After transfection with si-circ-PTK2, the level of E-cadherin mRNA was upregulated in both SK-OV-3 and OVCAR-3 cells (all *P* < 0.01), whereas the levels of Snail (*P* < 0.01) and vimentin (*P* < 0.01) mRNA were downregulated in SK-OV-3 cells, while only the level of Snail mRNA (*P* < 0.01) was downregulated in OVCAR3 cells (Fig. 7D). Therefore, this data indicates that the inhibition of circ-PTK2 can suppress the EMT pathway.

### Effects of circ-PTK2 on in vivo tumor growth of ovarian cancer

**Fig. 8:**
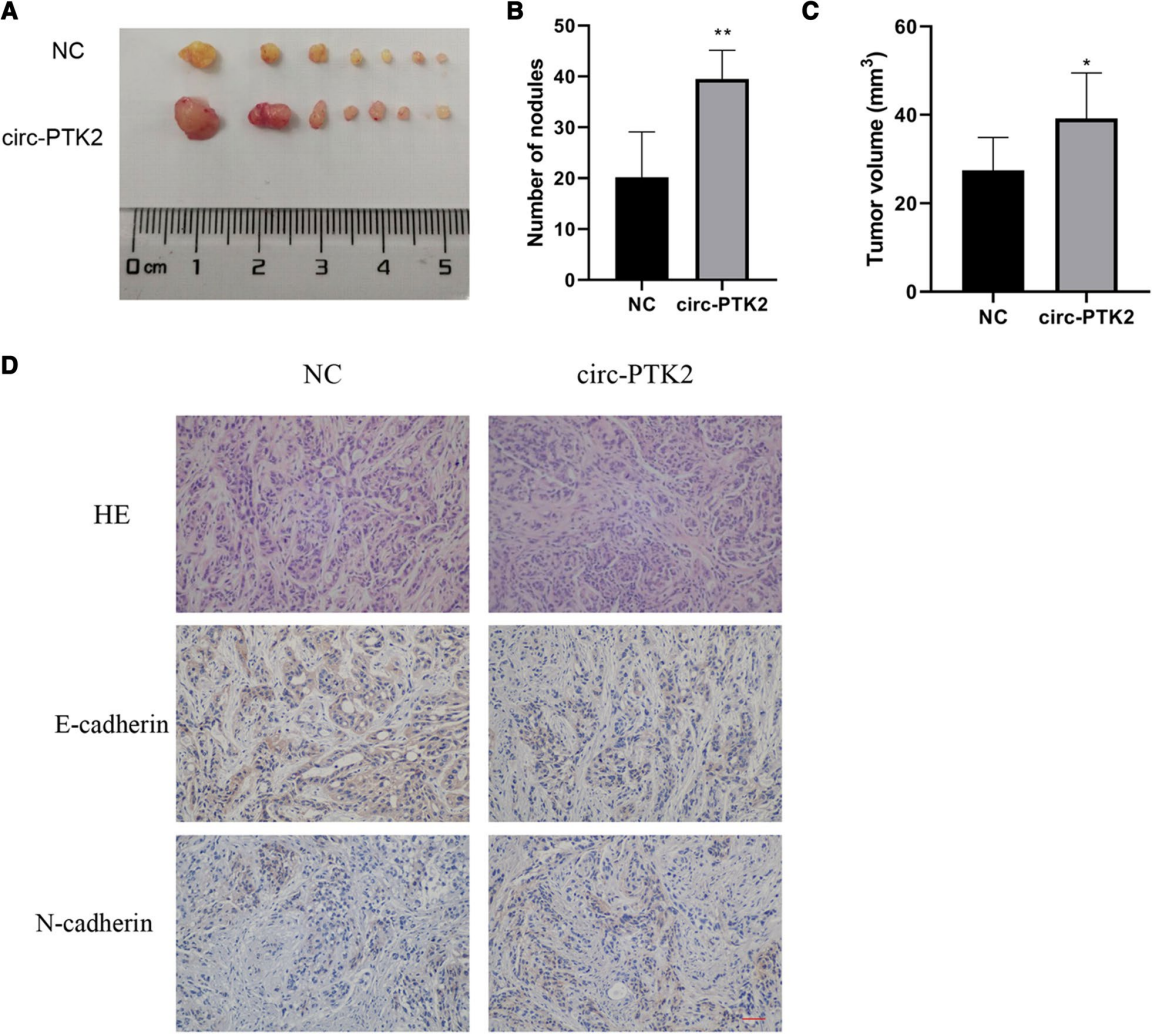
In vivo tumor formation assays validate the function of circ-PTK2 in tumorigenesis. **A** Tumors collected from euthanized nude mice. **B** Amounts of nodules present. **C** Tumor volume. **D** Hematoxylin and eosin staining of cellular structure and immunohistochemical staining to determine the protein level of E-cadherin and N-cadherin. Data are presented as the mean ± SD. *P < 0.05; **P < 0.01; ***P < 0.001

We used a tumor formation assay to investigate the functions of circ-PTK2 in vivo. The SK-OV-3 cells transfected with circ-PTK2 overexpression vector or vector alone were injected into nude mice. Tumors generated by the SK-OV-3 with circ-PTK2 overexpression presented comparatively larger volumes than those generated in the negative control group (SK-OV-3 cells transfection with control plasmids) (Fig. 8A). The circ-PTK2 overexpression group contained a larger number of nodules than those from the negative control group (*P* < 0.01) (Fig. 8B). The circ-PTK2 group also had a larger tumor volume than negative control group, which is consistent with the results in Fig. 6A (*P* < 0.05) (Fig. 8C). The tumor was then analyzed using HE and immunohistochemical staining, which showed that the cell number and density were greater in the circ-PTK2 overexpression group than those in the negative control group. The expression of E-cadherin was decreased, while that of N-cadherin was increased in the circ-PTK2 group compared with expression of these proteins in the negative control group (Fig. 8D). This is consistent with the results of the in vitro cell assays.

### miR-639 and FOXC1 cascade are targets of circ-PTK2 in ovarian cancer

**Fig. 9:**
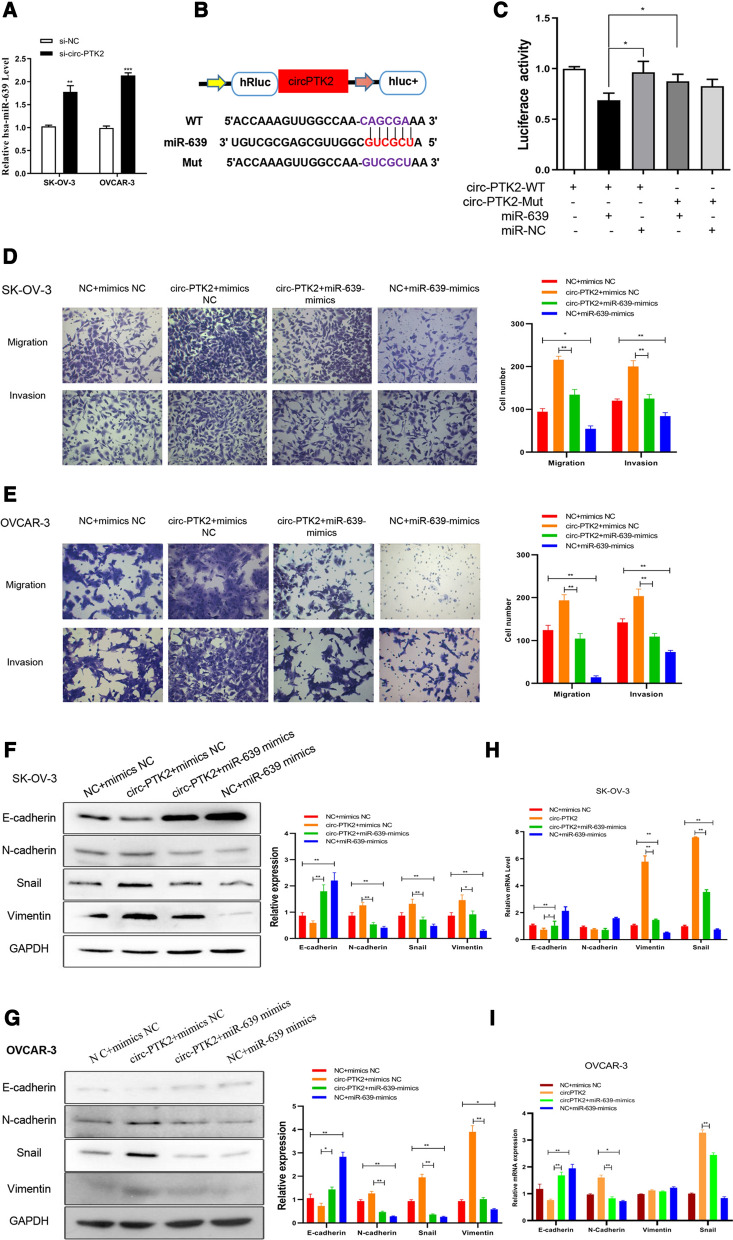
Validation of the interaction between circ-PTK2 and miR-639. **A** Reverse transcriptase-quantitative PCR validation of level of miR-639 following inhibition of circ-PTK2 expression. **B** Binding sequences between circ-PTK2 and miR-639 were predicted and wild-type and mutated circ-PTK2 genes were synthesized and cloned into luciferase vector to generate reporter. **C** Luciferase activity was determined to validate the interaction between circ-PTK2 and miR-639. **D** Transwell assay with SK-OV-3 cells showing enhanced cell migration and invasion due to overexpression of circ-PTK2, whereas overexpression of miR-639 inhibited those processes. **E** Transwell assay with OVCAR-3 cells showing enhanced cell migration and invasion due to overexpression of circ-PTK2, whereas overexpression of miR-639 inhibited those processes. **F** Western blot analysis of effect of overexpression of circ-PTK2 in SK-OV-3 cells: the protein level of E-cadherin was decreased, while the protein levels of N-cadherin, Snail, and vimentin were increased, although overexpression of miR-639 reversed the effect. **G** Western blot analysis of effect of overexpression of circ-PTK2 in OVCAR-3 cells: the protein level of E-cadherin was decreased, while the protein levels of N-cadherin, Snail, and vimentin were increased, although overexpression of miR-639 reversed the effect. **H** Overexpression of circ-PTK2 in SK-OV-3 cells decreased the mRNA level of E-cadherin and increased the mRNA level of vimentin and Snail, while overexpression of miR-639 reversed the effect; adjustment of expression of circ-PTK2 and miR-639 did not significantly affect expression of N-cadherin. **I** Overexpression of circ-PTK2 in OVCAR-3 cells decreased the mRNA level of E-cadherin and increased the mRNA level of N-cadherin and Snail, while that of miR-639 reversed the effect; adjustment of expression of circ-PTK2 and miR-639 did not significantly affect expression of vimentin. Data are presented as the mean ± SD. *P < 0.05; **P < 0.01; ***P < 0.001

Based on the principle of competing endogenous RNA (ceRNA), the interaction between circ-PTK2 and candidate miRNAs of circ-PTK2 was predicted and validated by RT-qPCR (Fig. 9A and Additional file 1: Fig. S1). We found that expression of miR-639 was negatively correlated with the inhibition of circ-PTK2 level by siRNA with increasing levels of miR-639 in both transfected SK-OV-3 (*P* < 0.01) and OVCAR-3 (*P* < 0.001) cell lines, which is consistent with the ceRNA principle (Fig. 9A). Based on the target sequences of miR-639 to circ-PTK2, a reporter was designed and conducted with luciferase reporter assay to validate if there was an interaction between circ-PTK2 and miR-639 (Fig. 9B). Reporters with circ-PTK2 target and mutated sequences were transfected into cells and assessed alongside the miR-639 mimics and miRNA mimic negative control (miR-NC). The luciferase activity was decreased when miR-639 mimics and circ-PTK2-WT reporter were used, but there was no significant change to the luciferase activity when either circ-PTK2-Mut or miR-NC was used, indicating that miR-639 can bind to circ-PTK2 (Fig. 9C). circ-PTK2 overexpression led to enhanced cell migration and invasion in both SK-OV-3 and OVCAR-3 cells; however, addition of miR-639 mimics reversed the outcome and suppressed cell migration and invasion (Fig. 9D, E). In SK-OV-3 and OVCAR-3 cells, circ-PTK2 + miR-639 mimics increased the mRNA and protein expression of E-cadherin but decreased the mRNA and protein expression of vimentin compared with expression in the circ-PTK2 + mimics NC group (Fig. 9F–I). Therefore, overexpression of miR-639 can reverse the EMT pathway activation induced by circ-PTK2 overexpression.

**Fig. 10:**
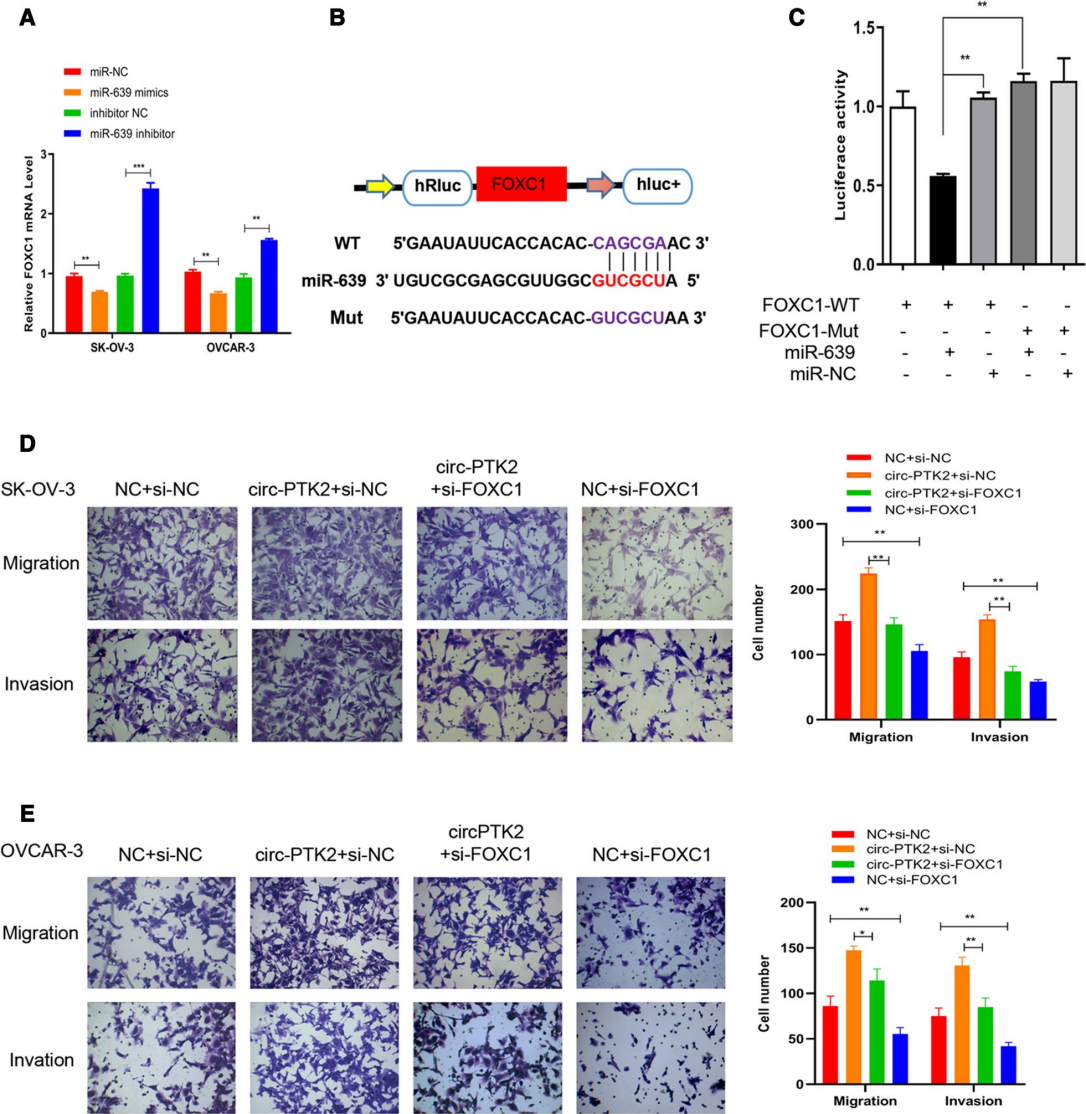
Validation of the interaction between miR-639 and FOXC1. **A** Reverse transcriptase-quantitative PCR validation of the level of *FOXC1* mRNA when miR-639 was inhibited or overexpressed. **B** Binding sequences between *FOXC1* mRNA and miR-639 were predicted, and wild-type and mutated *FOXC1* genes were synthesized and cloned into luciferase vector to generate a reporter. **C** Luciferase activity was determined to validate the interaction between *FOXC1* mRNA and miR-639. **D** Transwell assay with SK-OV-3 cells showing enhanced cell migration and invasion due to overexpression of circ-PTK2 while inhibition of *FOXC1* mRNA expression suppressed those processes. **E** Transwell assay with OVCAR-3 cells showing enhanced cell migration and invasion due to overexpression of circ-PTK2 while inhibition of *FOXC1* mRNA expression suppressed those processes. Data are presented as the mean ± SD. *P < 0.05; **P < 0.01; ***P < 0.001

In a similar manner, the mRNA targets of miR-639 were predicted. We found that expression of FOXC1 was downregulated when miR-639 mimics were transfected into SK-OV-3 and OVCAR-3 cells but was significantly upregulated when miR-639 inhibitor was used instead (Fig. 10A). The target sequence of miR-639 to FOXC1 mRNA was predicted and cloned to create a reporter for the luciferase reporter assay (Fig. 10B). The luciferase activity decreased to 50% in the FOXC1-WT + miR-639 mimic group than that in the control group. The luciferase activity showed no apparent change when either FOXC1-Mut or miR-NC was used, indicating that there is a valid interaction between miR-639 and FOXC1 mRNA (Fig. 10C). Furthermore, inhibition of FOXC1 mRNA expression by FOXC1-siRNA reversed circ-PTK2-enhanced cell migration and invasion in both SK-OV-3 and OVCAR-3 cells (all *P* < 0.01) (Fig. 10D, E).

## Discussion

In this study, we found that circ-PTK2 performs an important role in regulating cell migration and invasion in ovarian cancer. We found that circ-PTK2 expression was positively correlated to the level of CHD1L mRNA and that circ-PTK2 interacted with miR-639 and thereby regulated the expression of FOXC1.

In our previous study, we found that CHD1L expression in metastatic lesions of ovarian cancer was significantly higher than that in primary lesions of ovarian cancer [20]. The prognosis in patients with high CHD1L expression was inferior to those with low CHD1L expression [20], suggesting that CHD1L was involved in the development and metastasis of ovarian cancer. Similar results were found in those with breast cancer [23], non-small-cell lung cancer (NSCLC) [24], esophageal carcinoma [25], and pancreatic cancer [26]. Using the bioinformatics analyses, we found that the expression of CHD1L mRNA and protein in ovarian serous cancer was significantly higher than that in normal ovarian tissues, which was similar to our previous study based on tissue microarrays [20]. These results suggest that CHD1L is a potential oncogene for ovarian cancer, but the potential oncogenic mechanisms remains to be elucidated. In this study, we found that CHD1L can promote the migration and invasion of ovarian cancer cells through EMT. Using high-throughput sequencing, we found a circRNA circ-PTK2 that was positively correlated with CHD1L expression, which might be a novel biomarker and therapeutic target for ovarian cancer.

The PTK2 gene has been reported to express two types of circRNAs: circ_0003221 and circ_0008305. Both circRNAs have been correlated with cell proliferation and migration processes of cancer. In Xu et al. research, circ_0003221 was found highly expressed in migrated cells in metastatic lymph nodes. Silencing of circ-PTK2 inhibited the processes of cell proliferation and cell migration, whereas overexpression promoted proliferation and migration in bladder cancer. The high expression of circ_0003221 has also been correlated to poor clinicopathologic characteristics [27]. circ_0008305 expression has been correlated to inhibition of NSCLC. circ_0008305 overexpression augmented TIF1-gamma expression and inhibited TGF-beta-induced EMT and cell invasion during cancer. In NSCLC, circ_0008305 targeted miR-429/miR-200b-3p and inhibited their functions in activating the TGF-beta-induced EMT pathway by targeting TIF1-gamma [28]. In our study, circ-PTK2 (circ_0008305) promoted cell migration and invasion and angiogenesis processes, and circ-PTK2 expression was positively correlated to EMT conversion. Therefore, the functions of circ-PTK2 may be to those of circ_0003321 in bladder cancer, which acts as a cancer promoter. However, although it is the same circRNA, circ_0008305 is more likely a cancer inhibitor in NSCLC, but a cancer promoter in ovarian cancer. Therefore, although both circRNAs are derived from the same gene, their functions can vary in different cancer types.

In this study, we found that angiogenesis was strongly inhibited by knockdown of circ-PTK2, suggesting that circ-PTK2 also promote the progression of ovarian cancer by stimulating angiogenesis. The formation of a large number of microvessels is the basis for the development and metastasis of ovarian cancer [29]. Tumor blood vessels provide necessary oxygen and nutrients for tumor tissues, make them grow rapidly, and promote distant metastasis [30]. In recent years, anti-angiogenesis therapies are honored as one of the new treatment strategies for malignant tumors, especially for ovarian cancer and breast cancer [31, 32]. According to our study, promising therapies by targeting circ-PTK2 should also be investigated in the future to treat metastatic disease of ovarian cancer.

We validated miR-639 as the target of circ-PTK2. miR-639 expression was negatively correlated to cell migration and invasion, and overexpression via miRNA mimics inhibited both processes of cell migration and invasion in SK-OV-3 and OVCAR-3 cells. Based on our results, miR-639 presents as a tumor inhibitor in ovarian cancer. However, this is inconsistent with a previous report, where the expression of miR-639 was shown to be associated with the TGF-beta-induced EMT pathway, which activated metastasis in tongue squamous cell carcinoma by targeting FOXC1 [33]; this was also demonstrated in nasopharyngeal carcinoma. Wang et al. attempted to adjust the expression of miR-639 in nasopharyngeal carcinoma (NPC) C666-1 and NPC/HK1 cell lines and proved that overexpression of miR-639 promoted cell proliferation and migration, while suppression of miR-639 inhibited proliferation and migration in NPC [34]. Lei et al. demonstrated that miR-639 promoted cell proliferation and the cell cycle in human thyroid cancer and that the main function was performed by targeting CDKN1A [35]. Therefore, for most types of cancers so far reported, miR-639 acts as a cancer promoter. However, in ovarian cell lines, we increased the expression of miR-639 level by using corresponding miRNA mimics. This inhibited cell migration and invasion and altered the expression of EMT-relevant genes, whereby expression of E-cadherin was upregulated, while that of N-cadherin and Snail was downregulated. Therefore, a different miR-639 mechanism may operate in ovarian cancer than other types of cancer. Here circ-PTK2 expression was upregulated when cell migration and invasion were enhanced, indicating that miR-639 expression was downregulated, while the expression of the corresponding downstream target gene, FOXC1, was upregulated in ovarian cancer. We demonstrated that FOXC1 suppression inhibited cancerous cell migration and invasion and potential EMT. However, Wang et al. showed that high expression of FOXC1 can serve as a marker of benign ovarian tumors, enabling favorable prognosis for ovarian cancer [36], which is also inconsistent with our results. We hypothesize that the regulatory cascade of circ-PTK2, miR-639, and FOXC1 is valid. However, the occurrence of ovarian cancer and the level of miR-639 and FOXC1 may be regulated by various factors and not just circ-PTK2. In our study, we designed the experiments by adjusting the expression level of circ-PTK2 and determined the corresponding level of miR-639 and FOXC1 mRNA. However, ovarian cancer may be regulated by more than just the molecules described above. The regulatory network and underlying mechanism related to ovarian cancer are complicated, and various other genes and molecules may be involved in the network.

## Conclusion

Our results demonstrated a regulatory cascade related to circ-PTK2 expression, which potentially correlates to the processes of ovarian carcinoma. However, we are aware of the limitation of this study that a larger patient cohort is required to validate the association between circ-PTK2 expression and patient survival. In future studies, clinical specimens will be tested to determine the level of circ-PTK2 and miR-639 and FOXC1 in the disease state. In addition, more target genes and miRNAs of circ-PTK2 need to be assessed to enrich the corresponding regulatory network and help build a better understanding of the mechanism.

## Supplementary Information

**Additional file 1: Fig. S1.** qRT-PCR to validate the correlation between circ-PTK2 and candidate miRNAs.

## Data Availability

The datasets used and/or analyzed during the current study are available from the corresponding author on reasonable request.

## References

[1] Siegel R, Ma J, Zou Z, Jemal A (2014). Cancer statistics, 2014. CA Cancer J Clin.

[2] Goff BA, Mandel L, Muntz HG, Melancon CH (2000). Ovarian carcinoma diagnosis. Cancer.

[3] Urban N, Drescher C (2008). Potential and limitations in early diagnosis of ovarian cancer. Adv Exp Med Biol.

[4] Wright JD, Shah M, Mathew L, Burke WM, Culhane J, Goldman N, Schiff PB, Herzog TJ (2009). Fertility preservation in young women with epithelial ovarian cancer. Cancer.

[5] Suh KS, Park SW, Castro A, Patel H, Blake P, Liang M, Goy A (2010). Ovarian cancer biomarkers for molecular biosensors and translational medicine. Expert Rev Mol Diagn.

[6] Moss EL, Hollingworth J, Reynolds TM (2005). The role of CA125 in clinical practice. J Clin Pathol.

[7] Rastogi M, Gupta S, Sachan M (2016). Biomarkers towards ovarian cancer diagnostics: present and future prospects. Braz Arch Biol Technol.

[8] Hayes J, Peruzzi PP, Lawler S (2014). MicroRNAs in cancer: biomarkers, functions and therapy. Trends Mol Med.

[9] Esteller M (2011). Non-coding RNAs in human disease. Nat Rev Genet.

[10] Jeck WR, Sorrentino JA, Wang K, Slevin MK, Burd CE, Liu J, Marzluff WF, Sharpless NE (2013). Circular RNAs are abundant, conserved, and associated with ALU repeats. RNA.

[11] Warner JR (1999). The economics of ribosome biosynthesis in yeast. Trends Biochem Sci.

[12] Hansen TB, Jensen TI, Clausen BH, Bramsen JB, Finsen B, Damgaard CK, Kjems J (2013). Natural RNA circles function as efficient microRNA sponges. Nature.

[13] Chen LL, Yang L (2015). Regulation of circRNA biogenesis. RNA Biol.

[14] Salzman J, Chen RE, Olsen MN, Wang PL, Brown PO (2013). Cell-type specific features of circular RNA expression. PLoS Genet.

[15] He J, Xie Q, Xu H, Li J, Li Y (2017). Circular RNAs and cancer. Cancer Lett.

[16] Wang F, Nazarali AJ, Ji S (2016). Circular RNAs as potential biomarkers for cancer diagnosis and therapy. Am J Cancer Res.

[17] Burd CE, Jeck WR, Liu Y, Sanoff HK, Wang Z, Sharpless NE (2010). Expression of linear and novel circular forms of an INK4/ARF-associated non-coding RNA correlates with atherosclerosis risk. PLoS Genet.

[18] Zheng Q, Bao C, Guo W, Li S, Chen J, Chen B, Luo Y, Lyu D, Li Y, Shi G (2016). Circular RNA profiling reveals an abundant circHIPK3 that regulates cell growth by sponging multiple miRNAs. Nat Commun.

[19] Bao L, Zhong J, Pang L (2019). Upregulation of circular RNA VPS13C-has-circ-001567 promotes ovarian cancer cell proliferation and invasion. Cancer Biother Radiopharm.

[20] He WP, Zhou J, Cai MY, Xiao XS, Liao YJ, Kung HF, Guan XY, Xie D, Yang GF (2012). CHD1L protein is overexpressed in human ovarian carcinomas and is a novel predictive biomarker for patients survival. BMC Cancer.

[21] Rhodes DR, Yu J, Shanker K, Deshpande N, Varambally R, Ghosh D, Barrette T, Pandey A, Chinnaiyan AM (2004). ONCOMINE: a cancer microarray database and integrated data-mining platform. Neoplasia.

[22] Gyorffy B, Lánczky A, Szállási Z (2012). Implementing an online tool for genome-wide validation of survival-associated biomarkers in ovarian-cancer using microarray data from 1287 patients. Endocr Relat Cancer.

[23] Wang W, Wu J, Fei X, Chen W, Li Y, Shen K, Zhu L (2019). CHD1L promotes cell cycle progression and cell motility by up-regulating MDM2 in breast cancer. Am J Transl Res.

[24] Li Y, He LR, Gao Y, Zhou NN, Liu Y, Zhou XK, Liu JF, Guan XY, Ma NF, Xie D (2019). CHD1L contributes to cisplatin resistance by upregulating the ABCB1-NF-κB axis in human non-small-cell lung cancer. Cell Death Dis.

[25] Liu ZH, Zhang Q, Ding YJ, Ren YH, Yang HP, Xi Q, Cheng YN, Miao GL, Liu HK, Li CX (2017). Overexpression of CHD1L is associated with poor survival and aggressive tumor biology in esophageal carcinoma. Oncotarget.

[26] Liu C, Fu X, Zhong Z, Zhang J, Mou H, Wu Q, Sheng T, Huang B, Zou Y (2017). CHD1L expression increases tumor progression and acts as a predictive biomarker for poor prognosis in pancreatic cancer. Dig Dis Sci.

[27] Xu ZQ, Yang MG, Liu HJ, Su CQ (2018). Circular RNA hsa_circ_0003221 (circPTK2) promotes the proliferation and migration of bladder cancer cells. J Cell Biochem.

[28] Wang L, Tong X, Zhou Z, Wang S, Lei Z, Zhang T, Liu Z, Zeng Y, Li C, Zhao J (2018). Circular RNA hsa_circ_0008305 (circPTK2) inhibits TGF-β-induced epithelial-mesenchymal transition and metastasis by controlling TIF1γ in non-small cell lung cancer. Mol Cancer.

[29] Folkman J (2000). Incipient angiogenesis. J Natl Cancer Inst.

[30] Carmeliet P, Jain RK (2000). Angiogenesis in cancer and other diseases. Nature.

[31] Viallard C, Larrivée B (2017). Tumor angiogenesis and vascular normalization: alternative therapeutic targets. Angiogenesis.

[32] Markowska A, Sajdak S, Markowska J, Huczyński A (2017). Angiogenesis and cancer stem cells: new perspectives on therapy of ovarian cancer. Eur J Med Chem.

[33] Liu N, Chen NY, Cui RX, Li WF, Li Y, Wei RR, Zhang MY, Sun Y, Huang BJ, Chen M (2012). Prognostic value of a microRNA signature in nasopharyngeal carcinoma: a microRNA expression analysis. Lancet Oncol.

[34] Wang YH, Yin YW, Zhou H, Cao YD (2018). miR-639 is associated with advanced cancer stages and promotes proliferation and migration of nasopharyngeal carcinoma. Oncol Lett.

[35] Lei ST, Shen F, Chen JW, Feng JH, Cai WS, Shen L, Hu ZW, Xu B (2016). MiR-639 promoted cell proliferation and cell cycle in human thyroid cancer by suppressing CDKN1A expression. Biomed Pharmacother.

[36] Wang LY, Li LS, Yang Z (2016). Correlation of FOXC1 protein with clinicopathological features in serous ovarian tumors. Oncol Lett.

