# Supplementary figures and images for: circ-PTK2 (hsa_circ_0008305) regulates the pathogenic processes of ovarian cancer via miR-639 and FOXC1 regulatory cascade
Source: Cancer Cell Int. 2021 May 25;21:277. doi: 10.1186/s12935-021-01985-x (PMC8146250; doi:10.1186/s12935-021-01985-x)

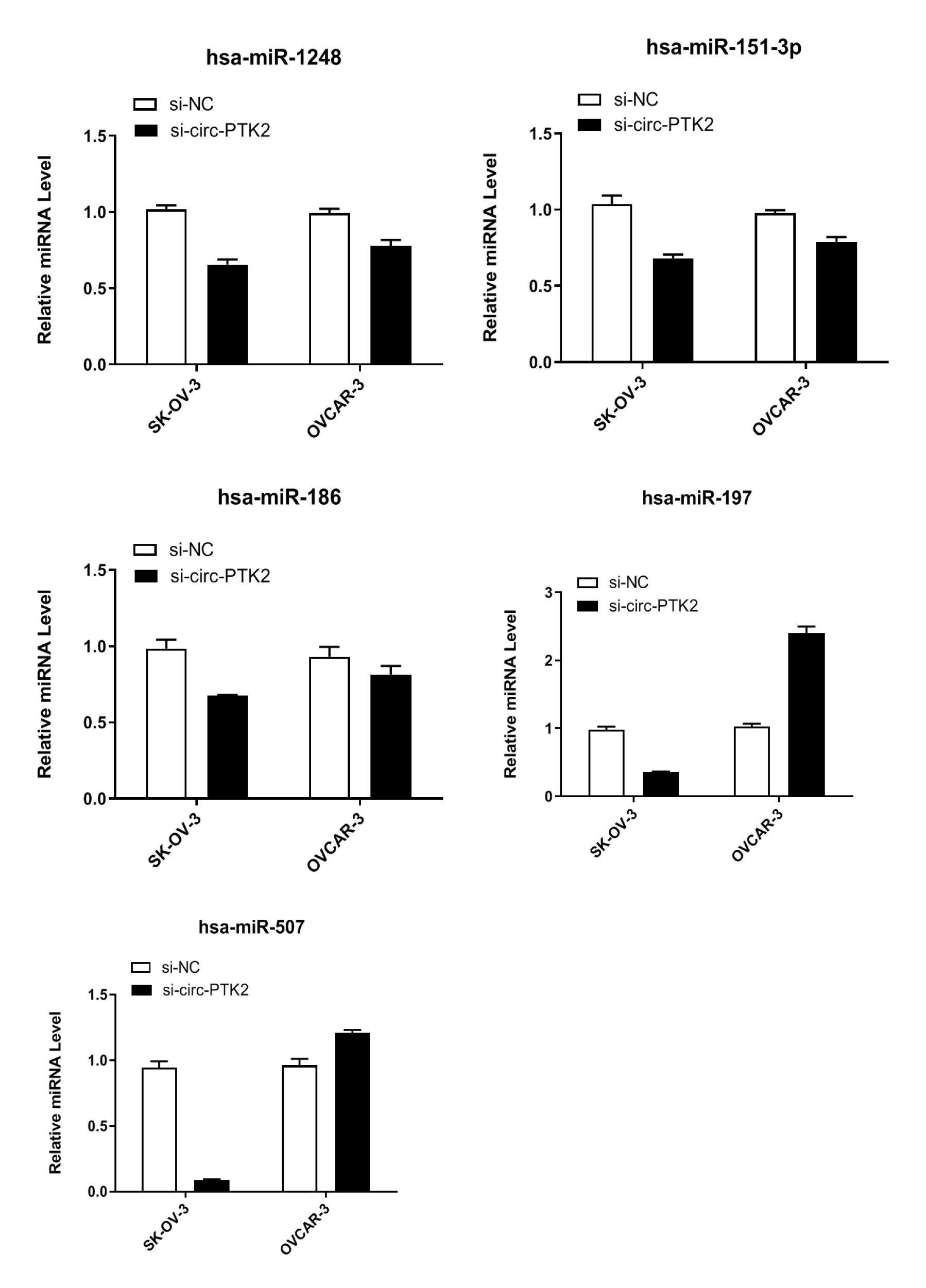

Supplement: Supplementary file 1 — Additional file 1: Fig. S1. qRT-PCR to validate the correlation between circ-PTK2 and candidate miRNAs. [file 12935_2021_1985_MOESM1_ESM.tif]
